# Identification of a psychiatric risk gene *NISCH* at 3p21.1 GWAS locus mediating dendritic spine morphogenesis and cognitive function

**DOI:** 10.1186/s12916-023-02931-6

**Published:** 2023-07-13

**Authors:** Zhi-Hui Yang, Xin Cai, Zhong-Li Ding, Wei Li, Chu-Yi Zhang, Jin-Hua Huo, Yue Zhang, Lu Wang, Lin-Ming Zhang, Shi-Wu Li, Ming Li, Chen Zhang, Hong Chang, Xiao Xiao

**Affiliations:** 1 Key Laboratory of Animal Models and Human Disease Mechanisms of the Chinese Academy of Sciences and Yunnan Province, Kunming Institute of Zoology, Chinese Academy of Sciences, Kunming, Yunnan China; 2 Kunming College of Life Science, University of Chinese Academy of Sciences, Kunming, Yunnan China; 3 Department of Blood Transfusion, The Second Affiliated Hospital of Kunming Medical University, Kunming, Yunnan China; 4 Department of Neurology, The First Affiliated Hospital of Kunming Medical University, Kunming, Yunnan China; 5 Clinical Research Center & Division of Mood Disorders, Shanghai Mental Health Center, Shanghai Jiao Tong University School of Medicine, Shanghai, China; 6 Shanghai Key Laboratory of Psychotic Disorders, Shanghai, China

**Keywords:** *Alu* element, Psychiatric disorders, *NISCH*, Dendritic spine morphogenesis, Working memory

## Abstract

**Background:**

Schizophrenia and bipolar disorder (BD) are believed to share clinical symptoms, genetic risk, etiological factors, and pathogenic mechanisms. We previously reported that single nucleotide polymorphisms spanning chromosome 3p21.1 showed significant associations with both schizophrenia and BD, and a risk SNP rs2251219 was in linkage disequilibrium with a human specific *Alu* polymorphism rs71052682, which showed enhancer effects on transcriptional activities using luciferase reporter assays in U251 and U87MG cells.

**Methods:**

CRISPR/Cas9-directed genome editing, real-time quantitative PCR, and public Hi-C data were utilized to investigate the correlation between the *Alu* polymorphism rs71052682 and *NISCH*. Primary neuronal culture, immunofluorescence staining, co-immunoprecipitation, lentiviral vector production, intracranial stereotaxic injection, behavioral assessment, and drug treatment were used to examine the physiological impacts of Nischarin (encoded by *NISCH*).

**Results:**

Deleting the *Alu* sequence in U251 and U87MG cells reduced mRNA expression of *NISCH*, the gene locates 180 kb from rs71052682, and Hi-C data in brain tissues confirmed the extensive chromatin contacts. These data suggested that the genetic risk of schizophrenia and BD predicted elevated *NISCH* expression, which was also consistent with the observed higher *NISCH* mRNA levels in the brain tissues from psychiatric patients compared with controls. We then found that overexpression of *NISCH* resulted in a significantly decreased density of mushroom dendritic spines with a simultaneously increased density of thin dendritic spines in primary cultured neurons. Intriguingly, elevated expression of this gene in mice also led to impaired spatial working memory in the Y-maze. Given that Nischarin is the target of anti-hypertensive agents clonidine and tizanidine, which have shown therapeutic effects in patients with schizophrenia and patients with BD in preliminary clinical trials, we demonstrated that treatment with those antihypertensive drugs could reduce *NISCH* mRNA expression and rescue the impaired working memory in mice.

**Conclusions:**

We identify a psychiatric risk gene *NISCH* at 3p21.1 GWAS locus influencing dendritic spine morphogenesis and cognitive function, and Nischarin may have potentials for future therapeutic development.

**Supplementary Information:**

The online version contains supplementary material available at 10.1186/s12916-023-02931-6.

## Background

Schizophrenia and bipolar disorder (BD) cause significant threats to public health and share clinical manifestations [1] and pathological features [2, 3]. For example, cognitive dysfunctions, such as impaired working memory, have been consistently observed in both schizophrenia and BD [4] and considered to be endophenotypes of both disorders [5–8]. Similar abnormalities in dendritic spine structure and density are also evident in the postmortem brain tissues of schizophrenics and BD patients [9–11]. Being highly heritable disorders, genetic overlap between schizophrenia and BD have also been demonstrated by epidemiological analyses and molecular genetic studies. For example, the offspring and siblings of schizophrenia have significantly higher risk of developing BD, and vice versa [12]; single nucleotide polymorphisms (SNPs) in the major histocompatibility complex (MHC) region, 3p21.1 region, *CACNA1C*, and *TRANK1* have shown genome-wide significant associations with both schizophrenia and BD [13, 14]. Notably, many of such genes associated with both disorders (e.g., *ZNF804A*, *NEK4*, *ANK3*, and *HOMER1/2*) have been demonstrated to affect dendritic spine morphogenesis [15–19], and some (such as *ZNF804A* [20, 21], *DRD2* [22–24], and *CACNA1C* [25, 26]) also influenced working memory in humans and mice. Therefore, if a gene showed significant associations with both schizophrenia and BD, it would also have putative effects on their common endophenotypes or etiological features. Identifying such genes and effects may provide clues for the underlying mechanisms for schizophrenia and BD.

Functional annotations of risk SNPs identified by genome-wide association studies (GWAS) have been widely applied to characterize risk genes for heritable disorders. Through integrative analyses by combining data of expression quantitative trait loci (eQTL) and GWAS, researchers found that majority of the risk SNPs were in the noncoding regions of the genome, and they likely affected mRNA expressions of distal genes [27, 28]. For example, integrative analyses found that a schizophrenia risk variant rs4420550 in 16p11.2 could regulate mRNA expression of *MAPK3* and *INO80E* through chromatin looping mechanisms [29]. Despite the prominent success of such integrative analyses, alternative methods are also appreciated in identifying risk factors for the disorders. Specifically, accumulating evidence showed that some functional variants can directly alter the mRNA expression of a gene or isoform [30], but they were not always detectable through the aforementioned integrative analyses. For instance, functional studies of a psychiatric risk variable number tandem repeat (VNTR) in 10q24.32 GWAS locus showed that it directly altered mRNA expression of an alternatively spliced isoform *AS3MT*^d2d3^ [31, 32], which was however not significant in the integrative analyses. Therefore, applications of both integrative and in-depth functional analyses are necessary to investigate the molecular underpinnings of psychiatric disorders.

Majority of the current genetic analyses focused on SNPs; nevertheless, growing efforts are also made on studying other sequence variations, such as VNTR, short tandem repeat (STR), and *Alu* short interspersed nuclear element (SINE), as they might be the functional units in psychiatric disorders [33–37]. For example, we and others reported the *Alu* polymorphisms showing strong LD with risk SNPs in many psychiatric GWAS loci [17, 33, 35, 37]. *Alu* polymorphisms are usually recognized as the presence or absence of an *Alu* element [38]. Given the longer DNA sequences (~ 300 bp) affected by these variants in the genome, *Alu* polymorphisms are presumed to exert greater impacts on the regulation of mRNAs and proteins than SNPs [39].

Therefore, we sought to characterize the risk sequence variations and genes shared between schizophrenia and BD using approaches combining integrative and in-depth functional analyses. We noticed that multiple indexed SNPs in the 3p21.1 genomic region showed significant associations with schizophrenia and BD [40]. One of these SNPs, rs2251219, exhibited genome-wide significant associations with both schizophrenia (*P* = 1.30 × 10^−15^, odds ratio = 1.062 for T-allele, 74,776 cases and 101,023 controls) and BD (*P* = 1.38 × 10^−10^, odds ratio = 1.063 for T-allele, 41,917 cases and 371,549 controls) in the recent GWAS [13, 14]. Remarkably, we previously reported a 335-bp functional *Alu* polymorphism rs71052682 in this genomic region, which is in strong LD with rs2251219 (*r*^2^ = 0.957 in Europeans [33] and *r*^2^ = 1.000 in Han Chinese) and showed significant regulatory effects on enhancer activities in U251 and U87MG cells [17]. We hence speculate that this *Alu* polymorphism (rs71052682) may be involved in both schizophrenia and BD through modulating gene expression in the 3p21.1 GWAS risk locus. However, it is still unclear which genes are directly affected by the *Alu* rs71052682 and what are their potential physiological impacts.

To address this question, we herein generate U251 and U87MG cells with different *Alu* polymorphisms through CRISPR/Cas9 editing, and reveal that the presence of the *Alu* element, which predicts a higher risk of schizophrenia and BD, increases the mRNA expression of *NISCH*. Notably, elevated expression of *NISCH* is also seen in the postmortem brain tissues from patients with either schizophrenia or BD compared with normal controls. We then show that higher expression of *NISCH* results in altered dendritic spine morphogenesis and impaired working memory, in concordant with the clinical manifestations in psychiatric patients. Intriguingly, treatment with antihypertensive drug clonidine could reduce the mRNA expression of *NISCH* and reverse the cognitive impairment caused by overexpressing *NISCH*. Our results thus describe a novel risk gene *NISCH* mediated by the human-specific *Alu* element in the 3p21.1 GWAS locus, and offer a potential new avenue into the therapeutic development in the clinical management of psychiatric patients.

## Methods

### Genome deletion of rs71052682 region by CRISPR/Cas9, off-target evaluation, and real-time quantitative PCR (RT-qPCR)

CRISPR/Cas9-based genome editing followed by RT-qPCR was performed to examine the molecular impact of rs71052682. Protospacer sequences of CRISPR/Cas9 against target genes were designed at https://cctop.cos.uni-heidelberg.de:8043/ [41]. We deleted the 561-bp DNA sequence containing rs71052682 by CRISPR/Cas9 with two sgRNAs encompassing the targeted loci. The sgRNA sequences were 5′-TGTATGTGAGGGTCGTTTCT-3′ (upstream) and 5′-AGAGGAAAACCAATACAGTT-3′ (downstream), respectively. The oligonucleotides were annealed and cloned into lentiCRISPR v2 plasmid (Addgene #52961), and the constructs were verified by Sanger sequencing. The DNA constructs expressing Cas9 and sgRNAs were co-transfected into HEK293T cells with psPAX2 and pMD2G plasmids to produce lentivirus.

U251 (human glioma), U87MG (human glioblastoma astrocytoma), HEK293T (human embryonic kidney 293T), and HeLa (human cervical cancer) cell lines were originally from the Cell Bank of Type Culture Collection of the Chinese Academy of Sciences and were cultured following our previous studies [29, 37]. About 50,000 cells of U251, U87MG, or HeLa were plated into 24-well plates before infection, and cells were infected with lentivirus expressing Cas9 and sgRNAs. Ninety-six hours after infection, the cells were treated with puromycin (1 μg/ mL) and then expanded in 6-well plates. HEK293T cells were cultured in 6-well plates and were transiently transfected with the lentiCRISPR v2 constructs using Lipofectamine 3000 at 50% confluency. Cells were treated puromycin (1 μg/ mL) and then expanded. Cells were harvested for genomic DNA purification followed by amplification of the targeted genome region, and Sanger sequencing was applied to confirm successful genome editing. The possible off-target effects of the sgRNAs were evaluated by T7EN1 assay. Twenty potential off-target genomic sites of the sgRNAs were predicted by CCTop (Additional file 1: Table S1). These DNA fragments were amplified (primers are shown in Additional file 1: Table S2), and the PCR products were then subject to T7EN1 cleavage assay.

RT-qPCR primers used for amplification of *NISCH* were 5′-AGTGTCCGCTTCTCAGCAAC-3′ (forward) and 5′-CCTTCAGGCTCCCACTCATC-3′ (reverse); primers for *NEK4* were 5′-AAAAAGAGGCTGAAAACCAACG-3′ (forward) and 5′-CTCCTCTCTCTGGCTGATAATGG-3′ (reverse); primers for *GNL3* were 5′-TGTGGGGAAAAGCAGCATTA-3′ (forward) and 5′-ATTGTGATCTGTTTGTCCAAGGG-3′ (reverse); primers for *PBRM1* were 5′-ATTCTCCTGAATATAAAGCCGCT-3′ (forward) and 5′-AGTCACTGTGCCCTGATTGTCT-3′ (reverse); primers for *GLT8D1* were 5′-GGCTGTTGCTCTCTTCTTACTGG-3′ (forward) and 5′-TCCTCTTGTCTCCCATCTACTGC-3′ (reverse). *RPS13* was used as the internal gene, and the primers used for the amplification of *RPS13* were 5′-CCCCACTTGGTTGAAGTTGA-3′ (forward) and 5′-CTTGTGCAACACCATGTGAA-3′ (reverse). Relative mRNA levels were presented as the means of 2^^–ΔCt^, and the protocols for RT-qPCR were described in our previous study [32].

### Public Hi-C datasets

We utilized public Hi-C data in dorsolateral prefrontal cortex (DLPFC) tissues to examine potential physical interactions between the *Alu* polymorphism rs71052682 and *NISCH* via the 3DIV website (http://www.3div.kr) [42] and 3D Genome Browser (http://3dgenome.fsm.northwestern.edu) [43, 44].

### Differential expression analyses of NISCH between psychiatric patients and controls

In order to confirm the correlation between *NISCH* and psychiatric diseases, we examined the mRNA expression of genes in the postmortem frontal and temporal cortex tissues of the PsychENCODE dataset, which included 559 schizophrenia patients, 222 BD patients, and 936 healthy controls [45]. Genes with transcripts per million reads (TPM) > 0.1 in at least 25% of samples were remained for further analyses. For differential expression analyses, hidden confounding factors were identified through surrogate variable analysis (SVA), and linear regression by covaring known biological, technical, and 4 SVs as fixed effects and subject-level technical replicates as random effects was conducted. Detailed information on tissue collection, RNA sequencing, gene expression quantification, and differential expression analysis was provided in the original study [45].

### Cell proliferation

Aberrant cell proliferation is thought to contribute to psychiatric disorders [46, 47]; we hence examined whether the *Alu* element at rs71052682 affected the cell proliferation rate. The Cell Counting Kit-8 (CCK-8) reagent (Beyotime Biotechnology, Haimen, China) was used to conduct cell proliferation tests. U251 cells with the genome deletion of rs71052682 or not were seeded into 96-well plates at a density of 1000 cells/well and cultivated for 24, 48, 72, 96, 120, and 144 h. Each well was added 10 μL of CCK-8 and then incubated for 1 h at 37 °C. Multidetection microplate reader (Bio-Rad Laboratories, Hercules, CA) was used to measure the optical density at 450 nm for each well.

### Animals

Wild-type C57BL/6J mice were purchased from Gem Pharmatech (https://www.gempharmatech.com/), and animals were kept in groups of 4 to 5 animals in transparent standard individually ventilated cages (IVC) with ad libitum access to food and water. The housing room was set to a 12-h light/dark cycle (lights on at 08:00 and lights off at 20:00), a temperature of about 22 °C. All experiments were approved by the Animal Ethics Committee of Kunming Institute of Zoology (NO: IACUC-RE-2021–11-001) and conformed to the National Advisory Committee for Laboratory Animal Research guidelines for ethical conduct in the care and use of animals.

### Cortical neurons culture, plasmid transfection, immunofluorescence staining, image capturing, and statistical analyses

Dendritic spines are crucial functional element in myriad neurons. Abnormal morphology and quantity of dendritic spines can affect synaptic functions, resulting in altered information transmission at the level of neural circuits [48–50]. There is also mounting evidence that abnormal dendritic spines are closely relevant to the occurrence of schizophrenia and BD [2, 3, 51]. We therefore investigated the impacts of *NISCH* on dendritic spine plasticity. Analyses of dendritic spine density were conducted following a previous study [52]. Briefly, pregnant C57BL/6J mice were anesthetized in transparent euthanasia chambers using compressed CO_2_ gas cylinders. About 5–8 mouse embryos (E16.5–17.5) were removed from the uterus and immediately transferred to 1 × HBSS with 1% penicillin–streptomycin (Life Technologies) on ice followed by washing 3 times with ice-cold HBSS without penicillin–streptomycin. The murine cortex was dissected from embryos in 1 × HBSS and then digested with 2 μg/mL Papain (Worthington, Cat. No: LS003119) and 5 U/mL DNase I (SIGMA, Cat. No: D4263-1VL) at 37 °C for 18 min. The neurons were seeded into 6-well plates with coverslips pre-coated by poly-d-lysine hydrobromide (SIGMA, Cat. No: P6407-5MG; 10 μg/mL) and cultured in a 2-mL culture medium containing Neurobasal (Gibco, Cat. No: 21103049), 2% B27 (Gibco, Cat. No: 17504044), 1 × GlutaMAX™-I (Gibco, Cat. No: 35050061), and 2.5% FBS (Biological Industries, Cat. No: 04–001-1ACS). At least 3 six-well plates of cultured neurons (8 × 10^5^ cells/well) were obtained from each pregnant mouse. Twelve hours later, the medium was replaced without FBS (Neurobasal, 2% B27, and 1 × GlutaMAX™-I). Cultures were incubated at 37 °C in a humidified, 5% CO_2_ atmosphere for 14 days.

Venus vectors (in order to clearly outline the dendritic spine) were transfected into the cultured neurons together with the pCDH constructs for *NISCH*-mCherry or control vectors (pCDH-GFP). Three days post-transfection, the neurons were fixed with 4% paraformaldehyde and 4% sucrose dissolved in PBS at room temperature, were stained with anti-mCherry (GeneTex, Cat. No: GTX128508) and GFP (Abcam, Cat. No: ab13970) overnight at 4 °C, and were randomly captured using an LSM 880 Basic Operation (Carl Zeiss) with Z-series image stacks scanning system (41 photos at 0.25 m intervals with a resolution of 1024 × 1024 pixels using a 100 objective and 10 digital zoom). In each batch, approximately 20 neurons were randomly photographed from more than 3 different coverslips, and the experiment was repeated 3 times. NeuronStudio [53] was used to analyze secondary or tertiary dendrites spines, including shape and density. At least 2 branches of each neuron were analyzed, and the number of a neuron’s dendritic spines was calculated as the average spine number of all its branches. For different types of dendritic spines, if a dendritic spine had a head and neck and the head width was more than 0.6 μm, it was classified as a mushroom spine; otherwise, it was a thin spine. When the dendritic spine had no discernible neck and the length to width ratio was less than 1, it was classified as a stubby spine. Two-tailed *t*-tests and two-way ANOVA followed by Bonferroni post hoc were conducted to estimate the differences in dendritic spine density between experimental groups.

### Co-immunoprecipitation (Co-IP)

We performed the Co-IP experiment to understand how Nischarin affected dendritic spines at the molecular level. HEK293T cells were seeded in a 100-mm cell culture dish. The cells were co-transfected with the plasmids pCDH-CMV-*NISCH*-FLAG-EF1-mCherry (overexpressing Nischarin with 3’FLAG) and pCAG-Actr2-HA (overexpressing Actr2 with 3’HA) using Lipo2000. After 72 h of transfection, the cells were rinsed with PBS and incubated for 30 min in a slow rate shaker at 4 °C with 1.5 mL IP lysis buffer (protein inhibitor was pre-added). The cell protein was obtained after 16,000 × *g* of centrifugation at 4 °C for 10 min. The protein was quantitated using BCA, 2% of the supernatant was used as the input sample, and about 2 mg (0.8 mL) of protein was taken as IP sample and IgG sample. The IP sample and IgG sample were respectively incubated with the corresponding antibody (#66006–2-Ig, Proteintech) and the same quantity of IgG (#B900620, Proteintech) overnight with a rotary mixer at 4 °C. The 2 samples were each incubated with 40 μL of Protein G Magnetic Beads (#10004D, Thermo Fisher Scientific) in the revolving mixer at 4 °C for 3–4 h on the second day. Magnetic beads were separated by magnetic stand and rinsed with pre-cooled IP lysis buffer for 4 times, once every 10 min. After the final wash, the lysis buffer was removed, and the magnetic beads were re-suspended with 100 µL 1 × SDS loading buffer and then incubated at 95 °C for 10 min. A 15-µL sample was loading to SDS-PAGE gel for western blot.

### Western blot

Many protein components, including postsynaptic density (PSD), SHANK postsynaptic scaffold complex, NMDA receptors, and AMPA receptors, play essential roles in dendritic spine morphology. We thus tested whether Nischarin had an effect on some of these key proteins. HT-22 cell line (mouse hippocampal neuronal cell line) was purchased from Procell Life Science & Technology Co., Ltd. (https://www.procell.com.cn/view/9174.html). Approximately 50,000 cells were seeded into 6-well plates for protein extraction. The cultured cells were rinsed once with PBS, and then lysed with RIPA solution buffer supplemented with 1 × PMSF. The adherent cells were scraped with cell scrapers and transferred to a pre-cooled centrifuge tube and placed in ice for 30 min. Every 5 min, the samples were vortexed for 10 s. Protein samples were collected from the supernatant following centrifugation at 4 °C for 10 min at 12,000 × *g*. Each protein sample was adjusted to the appropriate concentration after the protein concentration determination using the BCA protein assay kit (#23225, Thermo Fisher Scientific). 5 × loading buffer and β-mercaptoethanol were added to the sample, which was then incubated at 95 °C for 10 min. Protein samples were separated by 8% SDS-PAGE gel and then transferred to polyvinylidene fluoride (PVDF) membranes (#ISEQ00010, EMD Millipore). The nonspecific binding sites on the membrane were blocked with 5% skim milk at room temperature for 1 h, followed by overnighting incubation with the matching antibody (Psd95: #20665–1-AP, Proteintech; Grin2a: #19953–1-AP, Proteintech; Shank3: #64555, Cell Signaling Technology; Gapdh: #60004–1-IG, Proteintech) on the shaker at 4 °C. The secondary antibodies (#SA00001-1, Proteintech; #SA00001-2, Proteintech) were added to the blot for incubation after washing 3 times with TBST. After 1 h incubation at room temperature, the membrane was washed with TBST and proceeded to chemiluminescence.

### Lentiviral vector production and titration

Given that the *NISCH* gene’s coding sequences (CDS) exceed 5 kb, we packaged *NISCH* into lentivirus following previous studies [54, 55] and performed brain stereotaxic injection of *NISCH* virus into mice’s hippocampus to modulate its expression and analyze its impacts on murine behaviors. HEK293T cells were seeded into 15-cm plates and co-transfected with the envelope plasmid, packaging plasmid, and transfer vector (pCDH-CMV-MCS-EF1-GFP + Puro or pCDH-CMV-*NISCH*-EF1-mCherry + Puro). Ninety-six hours after transfection, the culture medium was collected and spined at 500 × *g* for 10 min at room temperature, passed through a 0.45-μm filter, and then transferred to a centrifugal tube for 2.5 h of superfast centrifugation with 25,000 rpm at 4 °C. The pellet was then resuspended with PBS.

To assess the titer of the lentivirus, HEK293T cells were cultured in 24-well plates and were incubated with the lentivirus in serial dilutions (1, 2.5, or 5 μL; the virus stock was diluted ten times) for 96 h. The cells were then treated with DNase for DNA extraction. The concentration of lentivirus was assessed by RT-qPCR with two pairs of primers: a lentiviral-specific transgene (WPRE gene: 5′-GTCCTTTCCATGGCTGCTC-3′ (forward) and 5′-CCGAAGGGACGTAGCAGA-3′ (reverse)) and a single copy gene-specific reference gene (Alb gene: 5′-TTTGCAGATGTCAGTGAAAGAGA-3′ (forward) and 5′-TGGGGAGGCTATAGAAAATAAGG-3′ (reverse)), following the standard procedure. The titer of the virus was obtained by the following formula: lentiviral copy number per cell = (copy number WPRE/copy number Alb) × 2; titer (TU/mL) = (number of cell seeded × lentiviral copy number per cell of the sample)/volume of used lentivirus (mL).

### Intracranial stereotaxic injections of lentivirus in adult mice

For stereotaxic injection, 7-week-old C57BL/6J mice were anaesthetized with isoflurane (3%) following by isoflurane (1%), and placed in a stereotaxic instrument (RWD Life Science). Lentivirus (5 × 10^9^ TU/mL, 0.5 µL for each site) was injected at two sites of the ventral CA1 (vCA1) of the hippocampus (anteroposterior [AP]: − 3.16 mm; mediolateral [ML]: ± 3.25 mm; dorsoventral [DV]: − 4.25 mm; − 3.25 mm). The eyes of the mice were coated with erythromycin eye ointment to prevent blindness caused by loss of blinking response to anesthetic. The operation involved making holes in the skull and injecting the virus to ensure no substantial bleeding. After the operation, the skin was sutured and disinfected promptly to prevent wound infection. For post-operative analgesic and anti-inflammatory effects, carprofen (5 mg/kg) and buprenorphine (0.1 mg/kg) were injected. Following this, mice were placed on a 37 °C constant temperature blanket until normal mobility was recovered. The mice were then returned to home cages and monitored. Three weeks after stereotaxic surgery, behavioral assessments were performed.

### Behavior paradigm

Male mice about 9 weeks old were grouped for behavioral tests. Mice were handled daily by a researcher for 1 week before the experiment in the behavioral test room (the temperature was maintained at 22 ± 1 °C). Mice were placed in the room for 1 h on the day of the experiment prior to the beginning of all tests. All behavioral tests were conducted during the dark/active phase of mice. The recording and data analysis system is provided by Shanghai Xinsoft Information Technology.

#### Open field test

The open field test was performed in a lidless opaque cube (40 cm × 40 cm × 40 cm). The bottom of the open field was evenly divided into 16 equal parts. The 4 parts in the middle were defined as the center, and the 4 parts in the corner were defined as corners. At the beginning of the test, mice were placed in the center and allowed to explore freely for 5 min. The time spent in the center and in the corners, as well as the total distance of mouse locomotion, was recorded for each mouse.

#### Elevated plus maze

The elevated plus maze consists of four perpendicular arms (5 cm × 35 cm), two closed arms with 15-cm-tall walls in the same straight direction, and two open arms with 0.5 cm tall walls to prevent mice from falling from the maze. The maze is 75 cm above the floor. Mice were placed in the center area of the maze, facing the open arm. Mice were allowed free access to all 4 arms for 5 min. The distance and time of the mice spent in the open and closed arms were recorded.

#### Y-maze

The Y-maze consists of three identical arms orientated at 120° angle from each other, and the arms were named A, B, and C during the test. Mice were placed at the center of the Y-maze and allowed to move undisturbed for 8 min. The mice were monitored, and their entry into three different arms consecutively is called spontaneous alternation behavior. The spontaneous alternation rate was calculated as spontaneous alternation/(total number of arm entries − 2).

### Treatment of cells and mice with antihypertensive drugs

Considering that Nischarin is a target of antihypertensive drugs, we investigated whether such drugs could affect *NISCH* expression and the abnormal murine behaviors caused by the dysregulation of this gene. About 50,000 U251 cells were plated into 24-well plates, and were treated with either DMSO or 0.1 mM clonidine (#C7897, Sigma-Aldrich) or 0.1 mM tizanidine (#T6950, Sigma-Aldrich) on the second day for 72 h. Total RNA was then harvested, reverse-transcribed into cDNA, and the expression level of *NISCH* was determined by RT-qPCR. The mice were fed with water containing either DMSO or 0.1 mM clonidine for 72 h before the behavioral experiments.

## Results

### Alu polymorphism rs71052682 increases mRNA expression of NISCH

We examined the allelic status of rs71052682 in multiple human cell lines, which revealed a homozygous presence of the *Alu* element in U251 and U87MG cells, and homozygous absence in SH-SY5Y, HEK293T, and HeLa cells. We previously showed that the presence of *Alu* element at rs71052682 increased the enhancer activities using luciferase reporter assays in U251 and U87MG cells. However, the *Alu* polymorphism rs71052682 is not covered in the public SNP-based eQTL datasets, and it is thus impossible to assess which gene was associated with rs71052682 using public datasets.

To examine the molecular impact of rs71052682, we applied CRISPR/Cas9 with dual sgRNAs to delete this *Alu* element as well as its flanking sequences in U251 and U87MG cells, and then examined the mRNA alterations of several genes. After CRISPR/Cas9-directed genome editing in cells, we examined 20 most likely off-target sites, and no obvious off-target cleavage with predicted bands size was observed (Additional file 1: Table S1 and Additional file 2: Fig. S1).

**Fig. 1:**
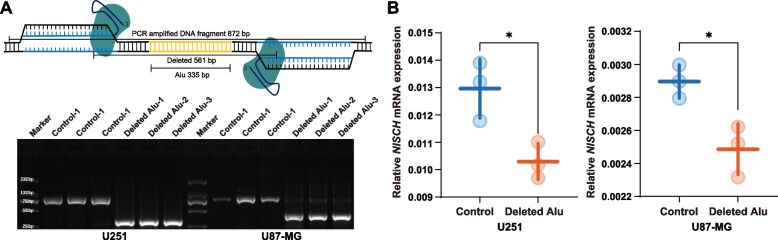
Deletion of genomic sequences spanning rs71052682 resulted in the downregulation of *NISCH* expression in U251 and U87-MG cells. **A** DNA fragment containing rs71052682 was knocked out using the CRISPR-Cas9 system. **B** Relative mRNA expression of *NISCH* in U251 and U87-MG cells. Data are shown as mean ± SD, and Student’s *t*-test was used to perform the significance test. *P < 0.05

Through RT-qPCR, we found that the mRNA level of *NISCH* was significantly reduced in both U251 and U87MG cells after the *Alu* and flanking sequences were deleted (Fig. 1). The mRNA levels of *NEK4*, *GNL3*, *PBRM1*, and *GLT8D1* were reduced after the deletion of the *Alu* element in U251 cells, but this trend was not reproduced in U87MG cells (Additional file 2: Fig. S2).

We also performed CRISPR/Cas9 editing in HEK293T and HeLa cells (the genotype at rs71052682 is “totally absent of *Alu* element”) using the same strategy to delete these flanking sequences around rs71052682 (i.e., 226-bp), and deletion of the flanking sequences in HEK293T and HeLa cells did not alter the expression of *NISCH* (Additional file 2: Fig. S3), suggesting that the observed reduced expression of *NISCH* after genome editing in U251 and U87MG cells was unlikely due to the flanking sequences.

### Chromatin contacts between Alu polymorphism rs71052682 and NISCH

Considering the distance between the *Alu* polymorphism rs71052682 and *NISCH* is as long as 180 kb and the *Alu* element at rs71052682 can affect *NISCH* expression, we then sought to test if there was a long-range chromatin interaction. We analyzed the public Hi-C data of the 3p21.1 region in DLPFC tissues in the 3DIV dataset and found extensive physical interactions between the DNA sequences spanning rs71052682 and *NISCH* (Additional file 2: Fig. S4) [42]. In addition, according to the Hi-C data in the DLPFC tissues in the 3D Genome Browser, rs71052682 and *NISCH* were also in the same large topologically associated domain (TAD) (Additional file 2: Fig. S5) [43, 44], further supporting putative regulatory effects of rs71052682 on *NISCH* expression in human brains.

### Higher expression of NISCH in psychiatric patients compared with controls

The above data collectively suggested the potential causative link between rs71052682 and *NISCH* mRNA expression. Since the presence of *Alu* element at rs71052682 was linked with the psychiatric risk T-allele at rs2251219, it is reasonable to hypothesize that higher mRNA expression of *NISCH* is associated with an increased risk of schizophrenia and BD. The PsychENCODE Consortium has analyzed the differentially expressed genes in the DLPFC tissues from 559 cases with schizophrenia and 222 cases with BD versus those from 936 controls [45]. Using these data, we found that the mRNA expression of *NISCH* was significantly higher in the DLPFC of patients with schizophrenia (*P* = 2.07 × 10^−9^) and patients with BD (*P* = 6.53 × 10^−4^) compared with unaffected controls [45], further confirming that higher expression of *NISCH* is linked with an increased risk of schizophrenia and BD. In addition, a recent transcriptome analysis, in the anterior cingulate cortex, also found that *NISCH* mRNA was significantly higher in 138 BD patients compared with 157 controls (*P* = 1.03 × 10^−3^) [56].

### Increased mRNA expression of NISCH affects dendritic spine morphogenesis

As stated above, schizophrenia and BD shared certain molecular and cellular neuropathology relevant to genetic risk effects. For example, dendritic spines are the postsynaptic compartments for the majority of excitatory synapses, and decreased density of dendritic spines has been observed in the prefrontal cortex of patients with schizophrenia and patients with BD compared with controls [10]. Many risk genes shared by schizophrenia and BD have been reported to affect dendritic spine morphogenesis, such as *NEK4*, *GNL3*, *PBRM1*, and *GLT8D1* [17, 57, 58]. Accordingly, if an identified risk gene could affect dendritic spine morphogenesis, it would further strengthen the conclusion of our analyses.

**Fig. 2:**
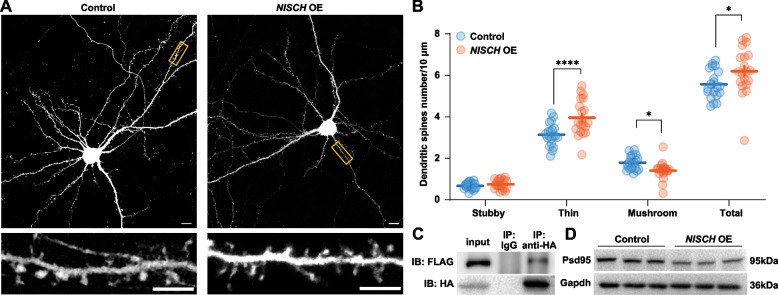
Overexpression of *NISCH* in primary cortical neurons affects dendritic spine morphogenesis. **A** Confocal images of whole neurons transfected with control or *NISCH* OE construct. Scale bars represent 20 μm. Dendritic branches were from each corresponding neuron, and scale bars represent 5 μm. Neuronal morphologies were visualized by staining for enhanced green fluorescent protein (EGFP). **B** Dendritic spine densities in the neurons transfected with control or *NISCH* OE construct. Results are presented as mean ± SEM (control, *n* = 20; *NISCH* OE, *n* = 20), and *P* < 0.05 after multiple corrections were defined as significant. All neuronal experiments were replicated at least twice with consistent conduct and acquisition parameters, and within each experiment, the dendritic spines were counted for each condition from more than three separate cultures. **C** Nischarin interacts with Actr2. pCDH-CMV-*NISCH*-FLAG-EF1-mCherry + Puro and the pCAC-Actr2-HA plasmid co-transfected into HEK293T cells. The cells were lysed for the immunoprecipitation (IP) experiment with an anti-HA antibody and the immunoblotting (IB) experiment with an anti-FLAG antibody after 72 h of transfection; 2% of the cell lysates were used as the input sample (top panel). Nischarin-FLAG and Actr2-HA were overexpressed in HEK293T cells (bottom panel). **D** Western blot analysis of Psd95 in HT22 cells overexpressing Nischarin. *NISCH* overexpression plasmid or control plasmid was transfected into HT22 cells for 72 h. Gapdh is used as an internal control. *P < 0.05, ***P < 0.0001. *NISCH* OE, *NISCH* overexpressed

We therefore tested whether overexpression of *NISCH* affected the density of dendritic spines in the primary cortical neurons. Differences in the total spine density between control neurons and those overexpressing *NISCH* were nominally significant (control, 5.57 ± 0.16 spines per 10 μm; overexpression of *NISCH*, 6.20 ± 0.26 spines per 10 μm, *F*_(19, 19)_ = 2.72, df = 38, *t* = 2.11, *P* = 0.041, two-tailed Student’s *t*-tests; Fig. 2A, B). Notably, in the neurons overexpressing *NISCH*, a significant decrease of the density of mushroom dendritic spines was observed (control, 1.79 ± 0.076 spines per 10 μm; overexpressing *NISCH*, 1.41 ± 0.095 spines per 10 μm, df = 114, *t* = 2.51, *P* = 0.041, multiple comparisons using Bonferroni correction in two-way ANOVA) with a simultaneously significant increase of the density of thin dendritic spines (control, 3.14 ± 0.12 spines per 10 μm; overexpressing *NISCH*, 3.96 ± 0.19 spines per 10 μm, df = 114, *t* = 5.41, *P* < 0.0001), while the densities of stubby spines were not significantly altered after overexpressing *NISCH* (control, 0.67 ± 0.036 spines per 10 μm; overexpressing *NISCH*, 0.75 ± 0.051 spines per 10 μm, df = 114, *t* = 0.53, *P* = 1.00).

Microstructure studies of dendritic spines have revealed a substantial network of actin microfilaments at the tip and neck of spines. The formation and dynamic regulation of dendritic spines are mainly controlled by filamentous actin (F-actin) and postsynaptic messengers [59, 60]. Previous studies have demonstrated that Nischarin interacted with LIMK1 to inhibit its activation and cofilin phosphorylation [61], both of which are important regulators of F-actin networks. Using Co-IP, we found that Nischarin interacted with actin-related protein 2 (Actr2) (Fig. 2C and Additional file 2: Fig. S6), one of the main components of the actin-related protein 2/3 complex (Arp2/3) that can bind F-actin to generate new branches. Therefore, Nischarin might modulate dendritic spine morphogenesis via interaction with Actr2.

In addition, altered morphogenesis of dendritic spines are thought to be related to postsynaptic structure. We therefore investigated whether Nischarin affected the expression of some key postsynaptic proteins in HT-22 cells. In line with the findings of reduced dendritic spines, *NISCH* overexpression significantly reduced the protein expression of Psd95 (major regulator of synaptic maturation) (Fig. 2D and Additional file 2: Fig. S7), while Grin2a and Shank3 were not affected. These findings suggest that Nischarin may influence postsynaptic structure by regulating the expression of Psd95.

### Elevated expression of NISCH impaired spatial working memory

**Fig. 3:**
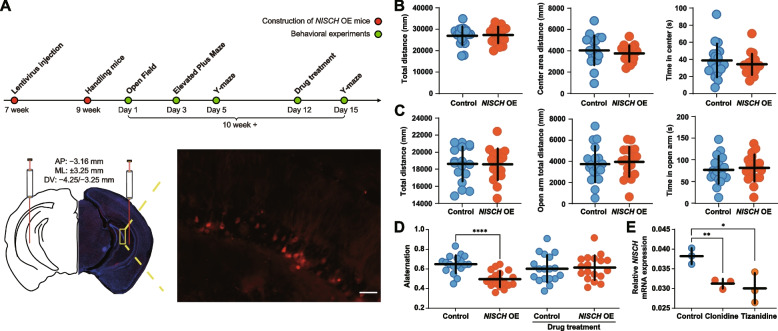
Overexpression of *NISCH* effects mouse behavior, and antihypertensive drugs rescued mice’s spatial working memory by affecting the *NISCH* expression. **A** Diagram of stereoscopic injection location and fluorescence expression in mouse brain. Scale bars represent 100 μm. **B** Open field test of *NISCH* OE mice and control mice. Measurement of total distance traveled, time in the center and distance in the center of the 5 min test. Data are shown as mean ± SD. *n* = 18 mice per genotype. **C** Elevated plus maze test of *NISCH* OE mice and control mice. Measurement of total distance traveled, time in the open arm, and distance in the open arm of the 5 min test. Data are shown as mean ± SD. *n* = 18 mice per genotype. **D** The spontaneous alternation score from the Y-maze test of *NISCH* OE mice and control mice and the Y-maze test of *NISCH* OE mice treated with clonidine for 3 days and control mice treated with DMSO for 3 days. Data are shown as mean ± SD.* n* = 18 mice per genotype. **E** U251 cells were treated with 0.1 mM clonidine or 0.1 mM tizanidine for 72 h to detect the effect of antihypertensive drugs on *NISCH* expression. Data are shown as mean ± SD. *P < 0.05, **P < 0.01, ****P < 0.0001. *NISCH OE*, *NISCH* overexpressed

To determine whether Nischarin has an impact on murine behaviors, *NISCH* overexpressing lentivirus was injected into the vCA1 of 7-week-old wild-type mice (Fig. 3A) followed by behavioral experiments. Locomotor activity, anxiety-like behavior, and spatial working memory were assessed.

In the open field test, we found that there was no significant difference between *NISCH* overexpressed (*NISCH* OE) mice and control mice in total distance (control, 26,995 ± 1023 mm distance; *NISCH* OE, 27,342 ± 911 mm distance, df = 34, *t* = 0.25, *P* = 0.80, *n* = 18–18; Fig. 3B), center area distance (control, 4044 ± 330 mm distance in the center; *NISCH* OE, 3748 ± 182 mm distance in center, df = 34, *t* = 0.79, *P* = 0.44, *n* = 18–18; Fig. 3B), and time (control, 38.68 ± 4.64 s in center; *NISCH* OE, 34.21 ± 2.93 s in center, df = 34, *t* = 0.82, *P* = 0.42, *n* = 18–18; Fig. 3B), suggesting intact locomotor activity of *NISCH* OE mice. In the elevated plus maze test, we did not observe a significant difference between *NISCH* OE mice and control mice in their total distance (control, 18,617 ± 481 mm distance; *NISCH* OE, 18,581 ± 437 mm distance, df = 34, *t* = 0.056, *P* = 0.96, *n* = 18–18; Fig. 3C) or the distance (control, 3736 ± 405 mm distance in open arm; *NISCH* OE, 3961 ± 337 mm distance in open arm, df = 34, *t* = 0.43, *P* = 0.67, *n* = 18–18; Fig. 3C) and time (control, 76.62 ± 7.56 s in open arm; *NISCH* OE, 81.17 ± 7.33 s in open arm, df = 34, *t* = 0.43, *P* = 0.67, *n* = 18–18; Fig. 3C) in the open arm.

Cognitive dysfunction is a common symptom of psychiatric disorders such as schizophrenia and BD, and several studies implicated the link between working memory and density of mushroom spines [62, 63]. Considering that *NISCH* plays a role in dendritic spine morphogenesis, we investigated whether this gene also affected working memory using Y-maze. We found that *NISCH* OE mice had a significantly lower spontaneous alternation rate compared with the control mice (control, 0.65 ± 0.021; *NISCH* OE, 0.50 ± 0.019, df = 34, *t* = 5.30, *P* < 0.0001, *n* = 18–18; Fig. 3D), suggesting that overexpression of *NISCH* impaired spatial working memory.

### Antihypertensive drugs rescued the effects of NISCH on murine spatial working memory

Accumulating evidence suggest that small molecules mediating dendritic spine morphogenesis may reveal novel drug targets for psychiatric disorders, and according to the druggable genome [64], Nischarin is a target of several approved small molecules and biotherapeutic drugs. For example, DrugBank [65] (http://www.drugbank.ca), Pharos [66] (https://pharos.nih.gov), and DGIdb [67] (https://www.dgidb.org/) prioritized Nischarin as a therapeutic target of clonidine (a long-established anti-hypertensive agent with presynaptic agonistic effect) and tizanidine (a fast-acting drug used for the management of muscle spasm) [68]. Several studies have found that antihypertensive drug treatment is associated with a lower risk of psychiatric disorders [69, 70]. Remarkably, clonidine has been shown to normalize sensorimotor gating deficits in patients with schizophrenia [71–73], and several studies have also demonstrated the effects of clonidine and tizanidine in BD patients during their manic phase [74–76]. These data suggest that Nischarin may have potentials for mechanistic studies and future therapeutic development. We hence tested whether these antihypertensive drugs could affect *NISCH* expression and found that clonidine significantly reduced the expression of *NISCH* in U251 cells compared with DMSO (*P* = 0.0071; Fig. 3E); similarly, another antihypertensive drug tizanidine had the consistent result (*P* = 0.032; Fig. 3E).

We further examined whether such antihypertensive drugs could alleviate the impairment of spatial working memory in mice caused by *NISCH* overexpression. Surprisingly, clonidine, a antihypertensive drug that targets *NISCH*, which did not affect the working memory in control mice (Additional file 2: Fig. S8), significantly elevated the spontaneous alternation percentage of *NISCH* OE mice in the Y-maze (control, 0.60 ± 0.033; *NISCH* OE, 0.61 ± 0.030, df = 34, *t* = 0.26, *P* = 0.80, *n* = 18–18; Fig. 3D). Therefore, reversing the elevated expression of *NISCH* using antihypertensive drug clonidine likely rescues the impaired spatial working memory in mice.

## Discussion

Large-scale GWASs significantly promoted our understanding of the overlapped genetic basis between psychiatric disorders, and several loci have been identified to show genome-wide significant associations with more than one psychiatric condition, such as 3p21.1. Nonetheless, there are many genes in this GWAS locus, and multiple genes are likely involved in the pathogenesis of psychiatric disorders. For instance, we previously reported *NEK4*, *GNL3*, and *PBRM1* as the psychiatric risk genes in the 3p21.1 region using integrative analyses based on DLPFC eQTL data, and manipulation of those risk genes could reduce mushroom dendritic spines. We also noted in the previous study that there might be additional psychiatric risk genes, which were linked to genetic risk independent of the above genes, at this GWAS locus [17]. In the present study, we show that deleting a functional *Alu* polymorphism in the 3p21.1 region led to the altered expression of *NISCH*, suggesting that this gene was likely mediated by the *Alu* element. Although the distance between *NISCH* and the *Alu* polymorphism was relatively long, chromatin interactions between them in brain tissues were confirmed using public Hi-C data. Intriguingly, the mRNA expression of *NISCH* was significantly higher in the postmortem brains of patients with psychiatric disorders compared with healthy controls. In line with our results, another recent study also demonstrated that *NISCH* was a risk gene shared between schizophrenia and BD through integrative analyses of GWAS and life course consistent methylation quantitative trait loci (meQTLs) datasets [77]. It is hence of great interest to explore the functional impact of this gene.

The function and molecular mechanisms of Nischarin in the brain, including its induced signal transduction pathways as well as the affected emotional disorders, have been extensively discussed in a recent review [78]. In their study, they also mentioned that Nischarin affected the formation of neuronal dendritic spines. Similarly, we showed that overexpression of *NISCH* in neurons resulted in a significantly lower density of mushroom dendritic spines and a simultaneously increased density of thin dendritic spines. It should be noticed that thin spines are referred to as “learning spines,” and mushroom spines are referred to as “memory spines,” whereas synaptic enhancement could lead to an enlargement of thin spines into mushroom spines [79, 80]. Our results thus suggest that psychiatric risk genes within the 3p21.1 region may participate in psychiatric illnesses through, at least in part, affecting dendritic spine morphogenesis. These findings are also in line with the previously reported abundant distribution of Nischarin in the F-actin-rich protrusions of neurons in the cerebral cortex [81, 82]. Previous studies have shown that Nischarin could regulate the p21-activated kinase (PAK1)-independent Rac1 signaling pathway [83] and negatively modulate the LIMK1/Cofilin pathway [61, 84]. Since both Rac1 and Cofilin affect actin dynamics, and members of the Rho GTPase family are actively involved in the morphological plasticity of dendritic spines in neurons, it is reasonable to hypothesize that this protein could regulate dendritic spine morphogenesis through the F-actin organization. Indeed, our data showed that Nischarin interacted with actin-related protein 2 (Actr2) and reduced Psd95 protein expression, which is consistent with the previous studies. In addition, given that PAK1 is a key regulator of cell proliferation, we also investigated whether the *Alu* polymorphism rs71052682 affected cell proliferation and found that deletion of rs71052682 caused a slower proliferation rate in U251 cells (Additional file 2: Fig. S9).

We report that the *Alu* insertion of rs71052682 at 3p21.1, which is linked with the psychiatric risk allele at rs2251219, leads to an increased expression of *NISCH*. In addition to schizophrenia and BD, rs2251219 also showed significant associations with an increased risk of major depression (*P* = 5.22 × 10^−6^, odds ratio = 1.015 for T-allele, 294,322 cases and 741,438 controls) [85] and higher anxiety levels (*P* = 3.34 × 10^−7^, beta = 0.011 for T-allele; GAD-7; *n* = 135,747) [86], despite the results were less significant. By contrast, rs2251219 did not show any evidence of association with attention deficit hyperactivity disorder (*P* = 0.416, odds ratio = 1.008 for T-allele, 38,691 cases and 186,843 controls) [87]. These data suggest that rs2251219 and *NISCH* might be primarily linked to risk of mood and psychotic disorders, which are often linked with altered dendritic spine morphogenesis and impaired working memory [4]. More intriguingly, according to the recent GWAS of general cognitive function (*n* = 282,014 individuals) [88] and intelligence (*n* = 269,867 individuals) [89], the psychiatric risk allele of rs2251219 showed genome-wide significant associations with worse cognitive abilities and lower intelligence test scores (both *P* < 5.00 × 10^−8^), which is also in line with our murine behavioral results. Therefore, this *Alu* polymorphism might modulate aberrant dendritic spine morphogenesis through increasing the expression of *NISCH*, which further leads to impaired working memory. However, cautions are also needed, as whether the aberrant dendritic spine morphogenesis caused by *NISCH* is sufficient to cause the cognitive symptoms in psychiatric disorders remains undetermined. Furthermore, neural circuits affected by *NISCH* are also unclear.

Notably, we found that the psychiatric risk T-allele at rs2251219 also exhibited significantly reduced systolic blood pressure (*P* = 1.26 × 10^−7^, beta =  − 0.164 for T-allele) and pulse pressure (*P* = 1.30 × 10^−14^, beta =  − 0.164 for T-allele) [90], as well as a decreased risk of hypertensive diseases (*P* = 1.55 × 10^−6^, odds ratio = 0.977 for T-allele) [91]. This indicates that the *Alu* polymorphism rs71052682 has a strong correlation with both neuronal and cardiovascular system, and such observation is also in line with the results showing that antihypertensive drugs had therapeutic potentials in the treatment of psychiatric disorders.

### Limitation

We acknowledge that a potential limitation of the present study is that the *Alu* element was not precisely deleted, which is due to the existence of multiple DNA sequences that are similar with the sequence harboring rs71052682 in the genome. The molecular impact of this *Alu* is hence not accurately quantified, and further precise deletion using the novel developed twin prime editing (twinPE) method may resolve this issue [92]. Second, while the stereotaxic injection of *NISCH* OE virus was performed in the mice’s hippocampus, the primary neuronal cultures were carried out using tissues from the prefrontal cortex, and the molecular biochemical experiments were conducted using multiple cell lines. Although our results suggest that *NISCH* might exert functions in multiple brain regions and cell types, further analyses involving tissues from the same brain region (e.g., hippocampus or mPFC) would strengthen the present study.

## Conclusions

Using a combinatory approach including functional genetics, neuronal culture, and behavioral assessments, we have reported a novel risk gene *NISCH* in the 3p21.1 psychiatric GWAS locus. The expression of this gene is dysregulated in the brains of schizophrenia patients and BD patients, and we have shown its effects on dendritic spine morphogenesis and cognitive function. More intriguingly, the cognitive dysfunction caused by the altered expression of *NISCH* can be rescued by antihypertensive drugs, providing a potential novel direction for future therapeutic approaches.

## Supplementary Information

**Additional file 1: Table S1.** Predicted potential off-target sites of the sgRNAs. **Table S2.** Primers used for testing the off-target effect of CRISPR/Cas9 during genome editing.

**Additional file 2: Fig. S1.** Off-target effect of CRISPR/Cas9 during genome editing was determined by T7EN1 assay. **Fig. S2.** Expression analysis of *NEK4*, *GNL3*, *PBRM1* and *GLT8D1* in U251 (A) and U87MG (B) cells after the *Alu* was deleted. **Fig. S3.** Expression analysis of *NISCH*, *NEK4*, *GNL3*, *PBRM1* and GLT8D1 in HEK293T (A) and HeLa (B) cells after the flanking sequence was deleted. **Fig. S4.** *Alu* element at rs71052682 physically interacts with *NISCH* in human DLPFC according to Hi-C data. **Fig. S5.** *Alu* element at rs71052682 and *NISCH* are located in the same topologically associated domain (TAD) in human DLPFC according to Hi-C data. **Fig. S6.** Nischarin interacts with Actin-related protein 2. **Fig. S7.** Overexpression of *NISCH* affects Psd95 protein levels. **Fig. S8.** Clonidine did not affect the spatial working memory of wild-type mice in the Y-maze. **Fig. S9.** Deletion of *Alu* element at rs71052682 effects U251 cell proliferation.

## Data Availability

All the GWAS data and statistical software used in this study were publicly available (which can be accessed through the following URLs), and all the generated results in this study were provided in the main text and supplemental data. URLs: Bipolar disorder GWAS: https://doi.org/10.6084/m9.figshare.14102594 BrainMeta: https://yanglab.westlake.edu.cn/data/brainmeta/cis_sqtl/ BrainSeq: http://eqtl.brainseq.org/phase2/eqtl/ 3DIV website: http://www.3div.kr 3D Genome Browser: http://3dgenome.fsm.northwestern.edu DrugBank: http://www.drugbank.ca Pharos: https://pharos.nih.gov DGIdb: https://www.dgidb.org/ CCTop: https://cctop.cos.uni-heidelberg.de:8043/ Gem Pharmatech: https://www.gempharmatech.com/ Procell Life Science & Technology Co., Ltd.: https://www.procell.com.cn/view/9174.html PsychENCODE: http://www.psychencode.org/ Schizophrenia GWAS: https://doi.org/10.6084/m9.figshare.19426775

## Abbreviations

BD: Bipolar disorder
CCK-8: Cell Counting Kit-8
CDS: Coding sequences
Co-IP: Co-immunoprecipitation
DLPFC: Dorsolateral prefrontal cortex
eQTLs: Expression quantitative trait loci
FDR: False discovery rate
GWAS: Genome-wide association studies
IVC: Individually ventilated cages
LD: Linkage disequilibrium
MHC: Major histocompatibility complex
PSD: Postsynaptic density
PVDF: Polyvinylidene fluoride
RT-qPCR: Real-time quantitative PCR
SINE: Short interspersed nuclear element
SNP: Single nucleotide polymorphism
STR: Short tandem repeat
SVA: Surrogate variable analysis
TAD: Topologically associated domain
TPM: Transcripts per million reads
twinPE: Twin prime editing
VNTR: Variable number tandem repeat
