# Supplementary material for: Identification of a psychiatric risk gene NISCH at 3p21.1 GWAS locus mediating dendritic spine morphogenesis and cognitive function
Source: BMC Med. 2023 Jul 13;21:254. doi: 10.1186/s12916-023-02931-6 (PMC10347724; doi:10.1186/s12916-023-02931-6)
Supplement: Supplementary file 1 — Additional file 1: Table S1. Predicted potential off-target sites of the sgRNAs. Table S2. Primers used for testing the off-target effect of CRISPR/Cas9 during genome editing. [file 12916_2023_2931_MOESM1_ESM.pdf]

Table S1. Predicted potential off-target sites of the sgRNAs.

sgRNAs		Coordinates	strand	MM	target_seq	PAM	location	gene name	gene id	PCR Size (bp)	Predicted Cleavage Size (bp)
Alu-1	On	chr3:52675699-52675721	-	0	TGTATGTG[AGGGTCGTTTCT]	AGG	intronic	PBRM1	ENSG00000163939	537/311	150/161
	off-1	chr10:73842440-73842462	-	3	TGAAGGTG[AGTGTCGTTTCT]	AGG	exonic	CAMK2G	ENSG00000148660	642	195/447
	off-2	chr1:22643118-22643140	+	3	TGCATGTG[AGGCTTGTTTCT]	GGG	intergenic	C1QC	ENSG00000159189	466	194/272
	off-3	chr19:42824033-42824055	-	3	TGGATGTG[AGGGGGGTTTCT]	GGG	intronic	AC004603.4	ENSG00000225877	727	212/515
	off-4	chr15:93985886-93985908	+	4	TGGACATG[AGGGTAGTTTCT]	GGG	intronic	LINC01580	ENSG00000258785	341	128/213
	off-5	chr10:69608363-69608385	-	3	TATTTGTG[TGGGTCGTTTCT]	GGG	intergenic	MTND1P20	ENSG00000226794	615	132/483
	off-6	chr7:90803137-90803159	+	3	AGTATTTG[GGGGTCGTTTCT]	TGG	intronic	CDK14	ENSG00000058091	726	220/506
	off-7	chr6:24192441-24192463	-	4	TCCATGTC[AGAGTCGTTTCT]	TGG	intronic	DCDC2	ENSG00000146038	557	103/454
	off-8	chr9:30567042-30567064	+	4	TGTCTTTG[TGGATCGTTTCT]	TGG	intronic	LINC01242	ENSG00000280683	577	255/322
	off-9	chr19:43388826-43388848	+	3	TGGATGTG[AGGGGGGTTTCT]	GGG	intergenic	CD177P1	ENSG00000204933	628	140/488
	off-10	chrX:134640323-134640345	+	4	TGACTGGG[AGGGTTGTTTCT]	GGG	intronic	RPS7P12	ENSG00000237432	483	147/336
Alu-2	On	chr3:52676248-52676270	+	0	AGAGGAAA[ACCAATACAGTT]	TGG	intronic	PBRM1	ENSG00000163939	537/311	150/161
	off-11	chr4:27005407-27005429	+	3	AGATGAAA[AATAATACAGTT]	GGG	intronic	STIM2	ENSG00000109689	594	72/522
	off-12	chr5:69930628-69930650	+	3	AAACGAAA[ACCAAAACAGTT]	TGG	intergenic	RP11-98J23.2	ENSG00000198237	846	261/586
	off-13	chr10:105477058-105477080	-	4	ACTGGATA[ACTAATACAGTT]	AGG	intergenic	RNU6-463P	ENSG00000207068	480	133/347
	off-14	chr13:72863305-72863327	+	3	ATAGGAAA[AGAAATACAGTT]	TGG	intronic	PIBF1	ENSG00000083535	676	144/532
	off-15	chr6:69596079-69596101	-	3	AATAGAAA[ACCAATACAGTT]	CGG	intergenic	AL358133.1	ENSG00000265362	844	339/505
	off-16	chr7:117567820-117567842	+	2	AGATGAAA[ACCAGTACAGTT]	TGG	intronic	CFTR	ENSG00000001626	831	372/459
	off-17	chr1:248152016-248152038	-	4	GCATAAAA[ACCAATACAGTT]	TGG	intergenic	OR2M5	ENSG00000162727	317	103/214
	off-18	chr1:25928446-25928468	-	4	TGGGGGAA[GCCAATACAGTT]	AGG	intergenic	AL033528.1	ENSG00000266763	555	170/385
	off-19	chr1:180059377-180059399	-	4	AGAAAATT[ACCAATACAGTT]	TGG	intronic	CEP350	ENSG00000135837	330	163/167
	off-20	chr16:31507234-31507256	-	4	TAAGGAAA[AAAAATACAGTT]	TGG	intronic	C16orf58	ENSG00000140688	444	175/269

Table S2. Primers used for testing the off-target effect of CRISPR/Cas9 during genome editing.

	Direction	Sequences (5'-3')
On	F	CAAGGTGTTGAAGTGGTGAAAG
	R	CTCATGCAACTTAATCACTATCACG
Off-1	F	GTCTCAGGCAAAGGCAGGTC
	R	GCATCTGGAACGCAGTAGGTG
Off-2	F	ACGAAGGAAGAGGCACCAATA
	R	GCATTTGCTTTGCCTGAGACT
Off-3	F	CTCTGCTTCCTGGTGCTGTTC
	R	AAATGGTGCCGTTGAGTCCT
Off-4	F	GCTTGCCCAGGGTGTTCTAT
	R	GTCCTCTGGCTGAAACTCACTAAC
Off-5	F	ATGTACTCGCAATGACTATGTGGA
	R	AGGTGGGAGGCTCGTTTG
Off-6	F	TGCTGTGCCTCATTTTCCATT
	R	TTGGGTGCCTTTTTGTCTCTC
Off-7	F	CGAATCACCCTACAGGACACC
	R	GGACTGGATACTCAATGGGCTT
Off-8	F	TTTCTTGCTTTCATAGCCCTTC
	R	ATTCTACCTGGTCTGAAAGTCTCC
Off-9	F	GCACCTGCTTGGCTGGATAC
	R	CTGAGTCTTTCACGGGAGCAC
Off-10	F	TGGCTATTGTGAGTAGTGCTGTTG
	R	AGGAGAAGGGAAGGACGGAA
Off-11	F	ATTGCCAGTGGTTTGCTCATA
	R	ACATGATGTCACAAAAACAGCCT
Off-12	F	TATCCTACCTTGGTGCTTGCTTA
	R	TTTCCCACCCGCTACTCTCA
Off-13	F	TGGGCTGAGACAATGGGGT
	R	ACAGGTTCTGGAGTCCCTTTCAT
Off-14	F	CAAAGGACAGACAGCAAAGCAA
	R	CCCTCTTGGAAATCTCACACG
Off-15	F	CAGGCAGATGAAAGAGTATGTAGAC
	R	TGTTTGGGTTATGGGGGTG
Off-16	F	GCTCTTGAAATGGCAGATTGTAT
	R	GCCCCCAGTGTAAAGTTCAGTC
Off-17	F	GCTGAGATTCAAGGGCTGCTAT
	R	ACCACAACCAACACAAAAAGGA
Off-18	F	CCCAGTCTTACCCCTTTCCAT
	R	TAAATGTGCTTCTGTGCTGTTGAC
Off-19	F	CACAGGTCTGGAAGGTCAAAACTA
	R	CGAGCAAAGATGTCTGATTATGG
Off-20	F	TGGCAATAACGACAAAGAAACC
	R	CCCTCCCCTTTATCCCTATTC
