# Supplementary material for: Identification of a psychiatric risk gene NISCH at 3p21.1 GWAS locus mediating dendritic spine morphogenesis and cognitive function
Source: BMC Med. 2023 Jul 13;21:254. doi: 10.1186/s12916-023-02931-6 (PMC10347724; doi:10.1186/s12916-023-02931-6)

Figure S1. Off-target effect of CRISPR/Cas9 during genome editing was determined by T7EN1 assay. These data showed no CRISPR/Cas9 off-target effect in our study.

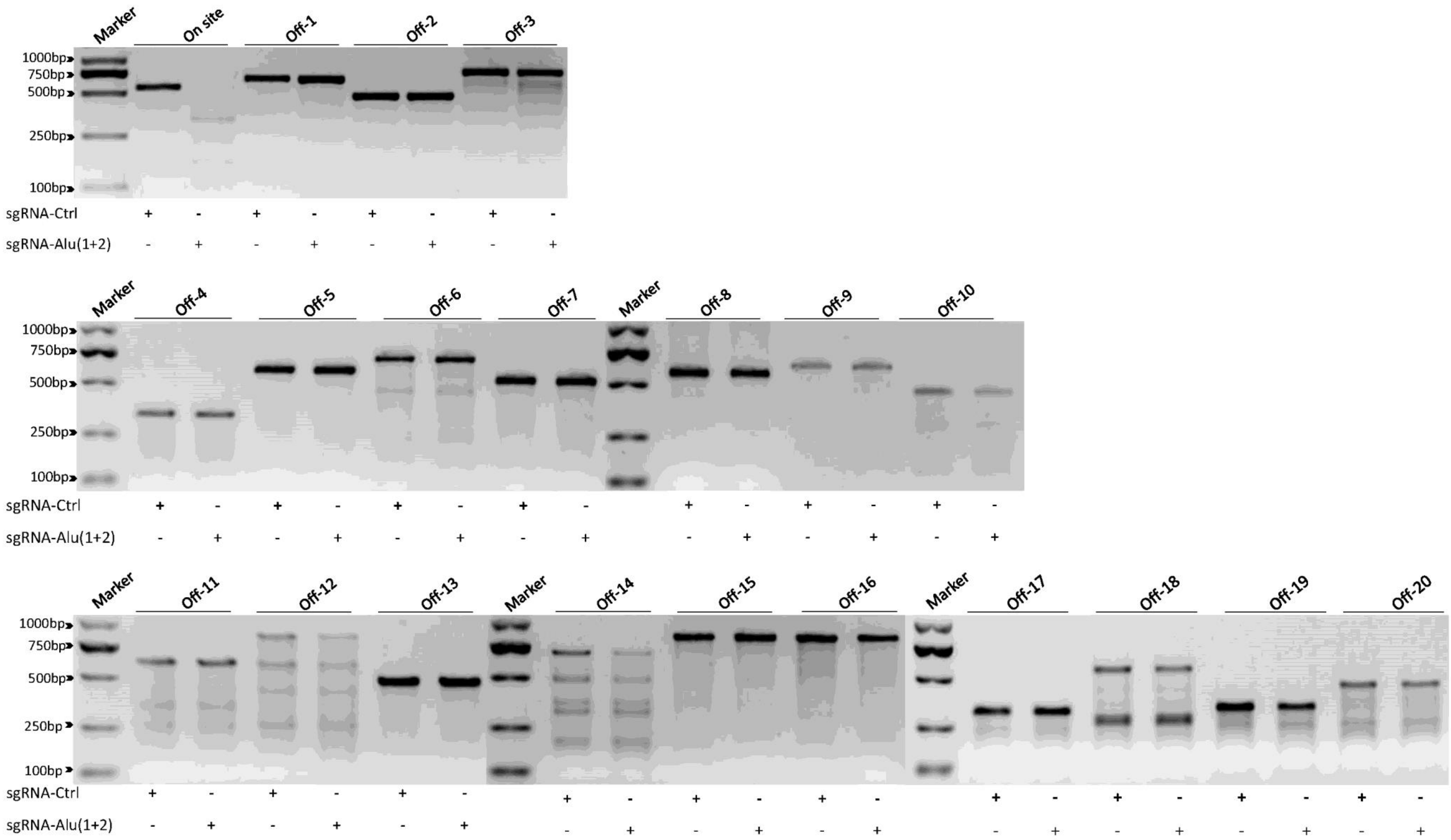

Figure S2. Expression analysis of *NEK4*, *GNL3*, *PBRM1* and *GLT8D1* in U251 (A) and U87MG (B) cells after the *Alu* was deleted. Error bars indicated standard deviation. **P* < 0.05, ***P* < 0.01.

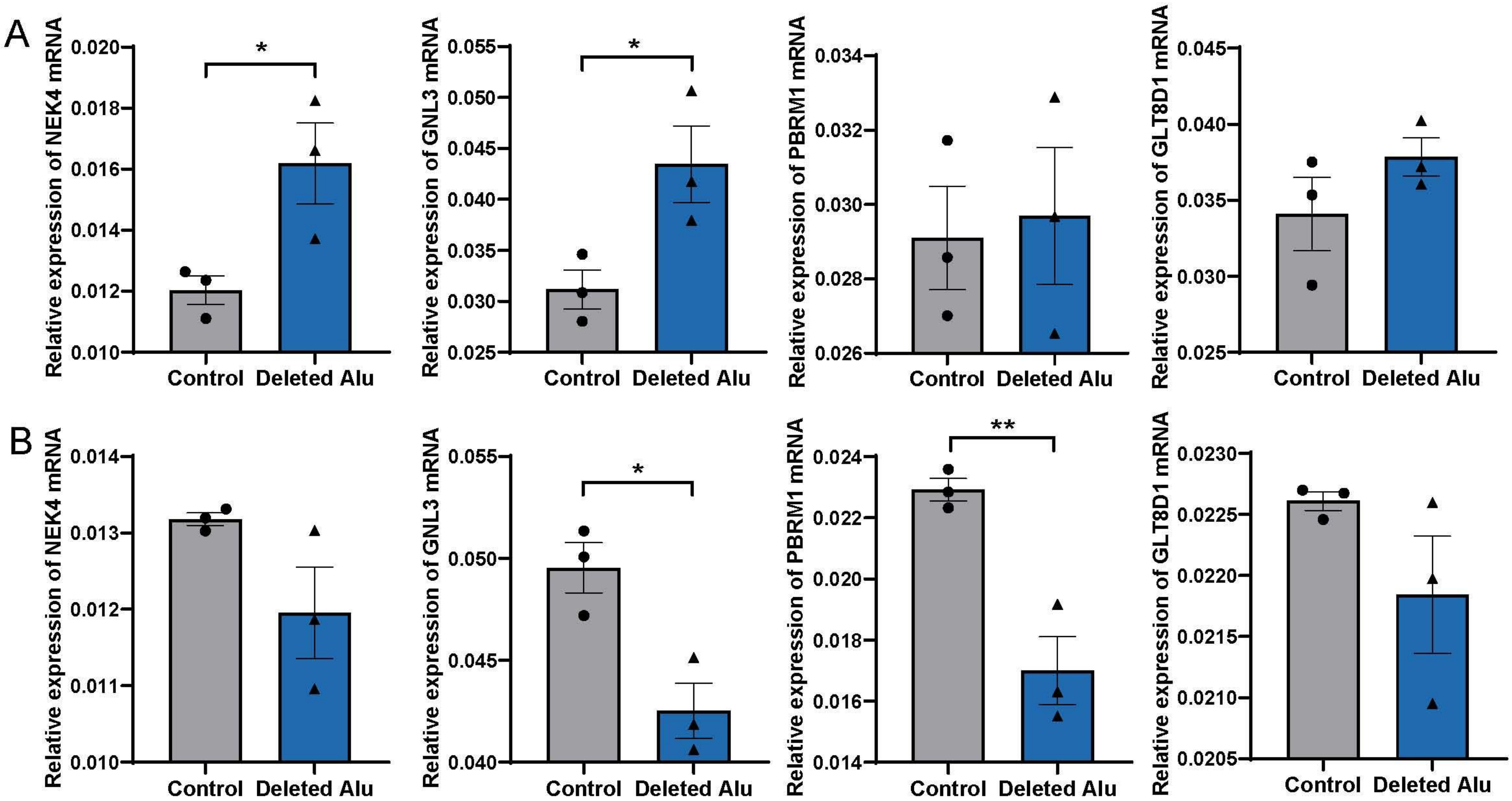

Figure S3. Expression analysis of *NISCH*, *NEK4*, *GNL3*, *PBRM1* and *GLT8D1* in HEK293T (A) and HeLa (B) cells after the flanking sequence was deleted. Error bars indicated standard deviation. *$P < 0.05$, **$P < 0.01$.

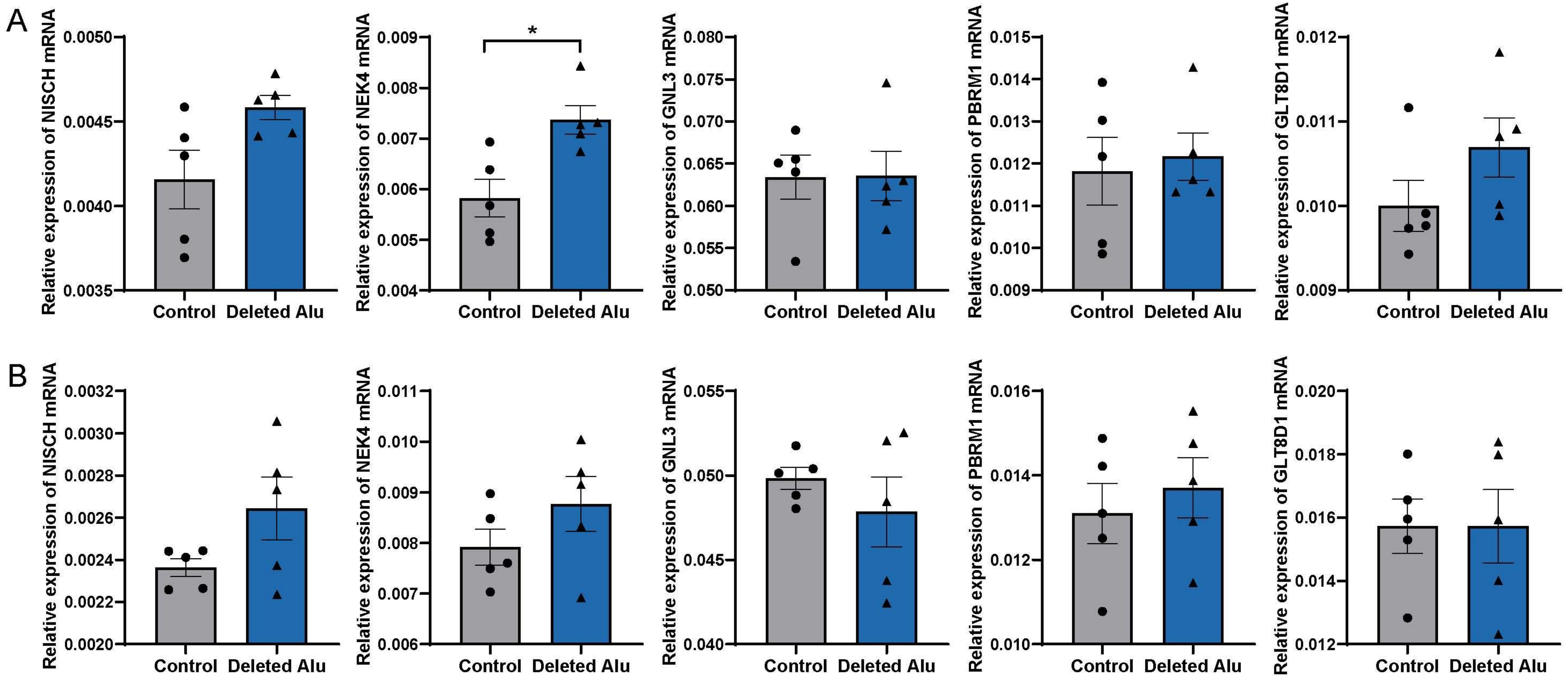

Figure S4. ***Alu*** **element at rs71052682 physically interacts with** ***NISCH*** **in human DLPFC according to Hi-C data.** Visualization was performed using the 3DIV website (http://www.3div.kr).

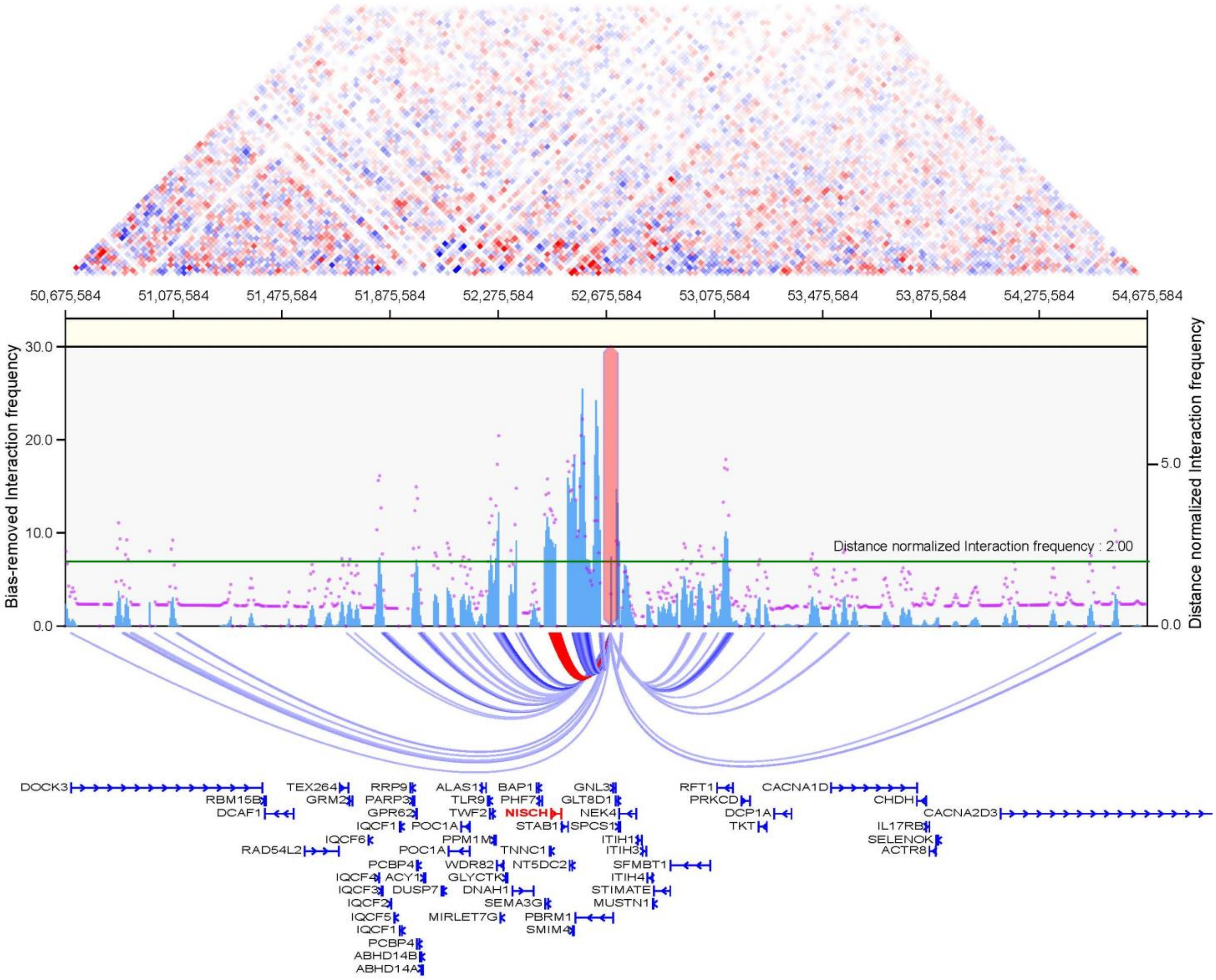

Figure S5. ***Alu*** **element at rs71052682 and** ***NISCH*** **are located in the same topologically associated domain (TAD) in human DLPFC according to Hi-C data.** Visualization was performed using the 3D Genome Browser in human genome (hg38) with a resolution of 40-kb.

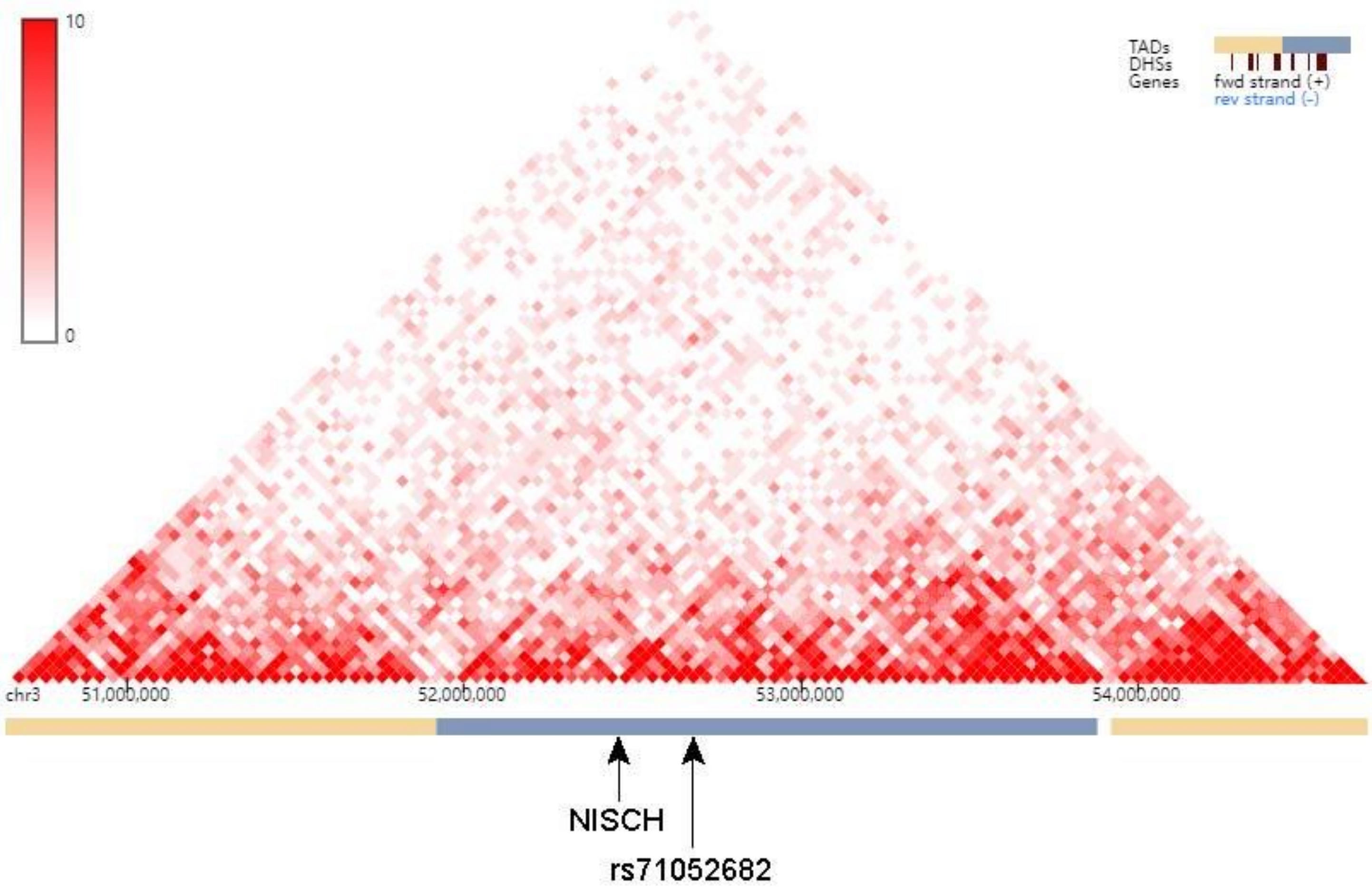

Figure S6. Nischarin interacts with Actin-related protein 2. (A) Western blot with anti-FLAG antibody to detect ACTR2 (HA) and Nischarin (FLAG) interaction from immunoprecipitation samples using IgG and HA antibodies. (B) Western Blot with anti-HA antibody detects ACTR2 (HA) plasmid expression and protein enrichment during immunoprecipitation (B). The cell lysates (input) were also blotted with anti-FLAG (A) or anti-HA (B).

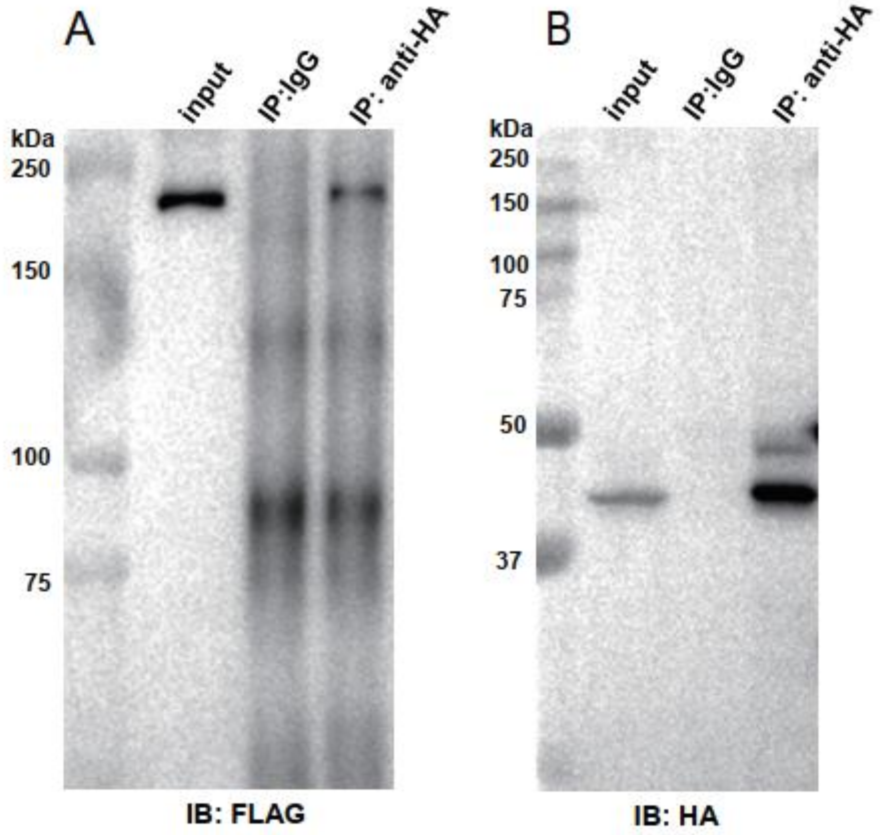

Figure S7. Overexpression of *NISCH* affects Psd95 protein levels. Western blot was used to detect the expression of Psd95 (A), Grin2a (B) and Shank3 (C) in *NISCH* overexpressed or control HT-22 cells. Gapdh was used as an internal reference (D). NISCH OE: NISCH overexpressed.

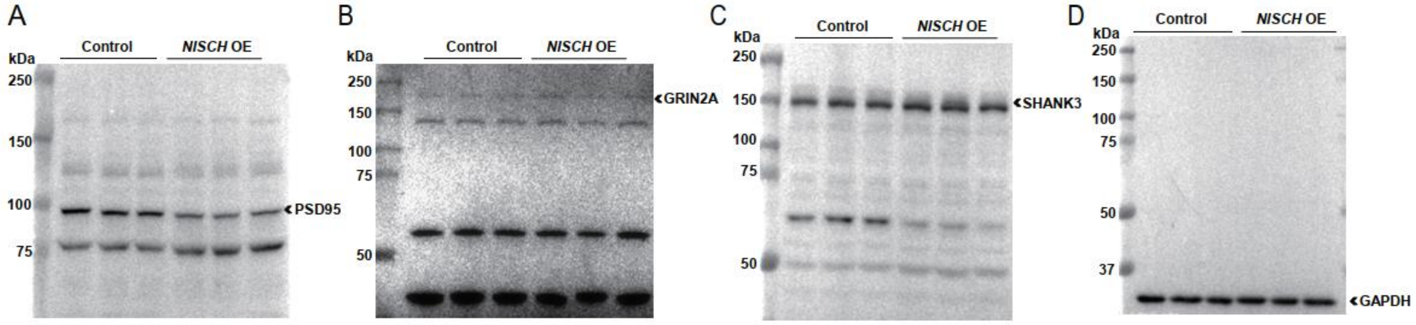

Figure S8. Clonidine did not affect the spatial working memory of wild-type mice in the Y-maze. 8-week-old wild-type mice were fed with 0.1 mM clonidine water or DMSO water (DMSO volume added to water is equivalent to clonidine volume) for 72 hours before conducting the Y-maze test. Y-maze test alternation score was calculated. The data are presented as mean with SEM, and the significance test was performed using the student's t-test.

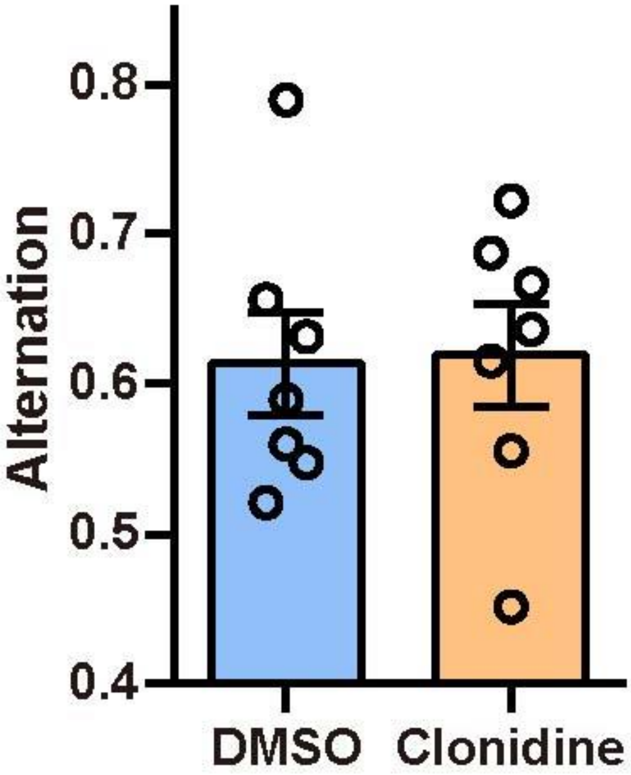

Figure S9. Deletion of *Alu* element at rs71052682 effects U251 cell proliferation. Cell Counting Kit-8 was used to detect optical density at 450 nm for U251 cells with or without CRISPR-Cas9 deletion of *Alu* element at rs71052682 at the same time every day. The results are presented as mean ± SEM (control, $n = 4$; deletion of *Alu* element at rs71052682, $n = 4$), with a two-way ANOVA for statistical analysis. $^{**}P < 0.01$, $^{****}P < 0.0001$.

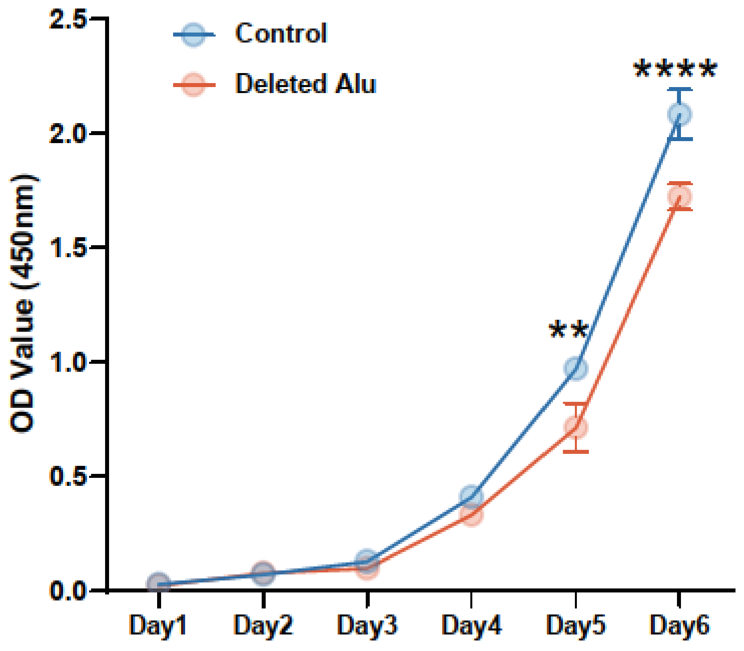

Supplement: Supplementary file 2 — Additional file 2: Fig. S1. Off-target effect of CRISPR/Cas9 during genome editing was determined by T7EN1 assay. Fig. S2. Expression analysis of NEK4, GNL3, PBRM1 and GLT8D1 in U251 (A) and U87MG (B) cells after the Alu was deleted. Fig. S3. Expression analysis of NISCH, NEK4, GNL3, PBRM1 and GLT8D1 in HEK293T (A) and HeLa (B) cells after the flanking sequence was deleted. Fig. S4. Alu element at rs71052682 physically interacts with NISCH in human DLPFC according to Hi-C data. Fig. S5. Alu element at rs71052682 and NISCH are located in the same topologically associated domain (TAD) in human DLPFC according to Hi-C data. Fig. S6. Nischarin interacts with Actin-related protein 2. Fig. S7. Overexpression of NISCH affects Psd95 protein levels. Fig. S8. Clonidine did not affect the spatial working memory of wild-type mice in the Y-maze. Fig. S9. Deletion of Alu element at rs71052682 effects U251 cell proliferation. [file 12916_2023_2931_MOESM2_ESM.pdf]
